# Supplementary material for: Fermentation of Jamaican Cherries Juice Using Lactobacillus plantarum Elevates Antioxidant Potential and Inhibitory Activity against Type II Diabetes-Related Enzymes
Source: Molecules. 2021 May 12;26(10):2868. doi: 10.3390/molecules26102868 (PMC8151855; doi:10.3390/molecules26102868)
Supplement: Supplementary file 1 [file molecules-26-02868-s001.zip › molecules-1212451-supplementary.pdf]

Supplementary

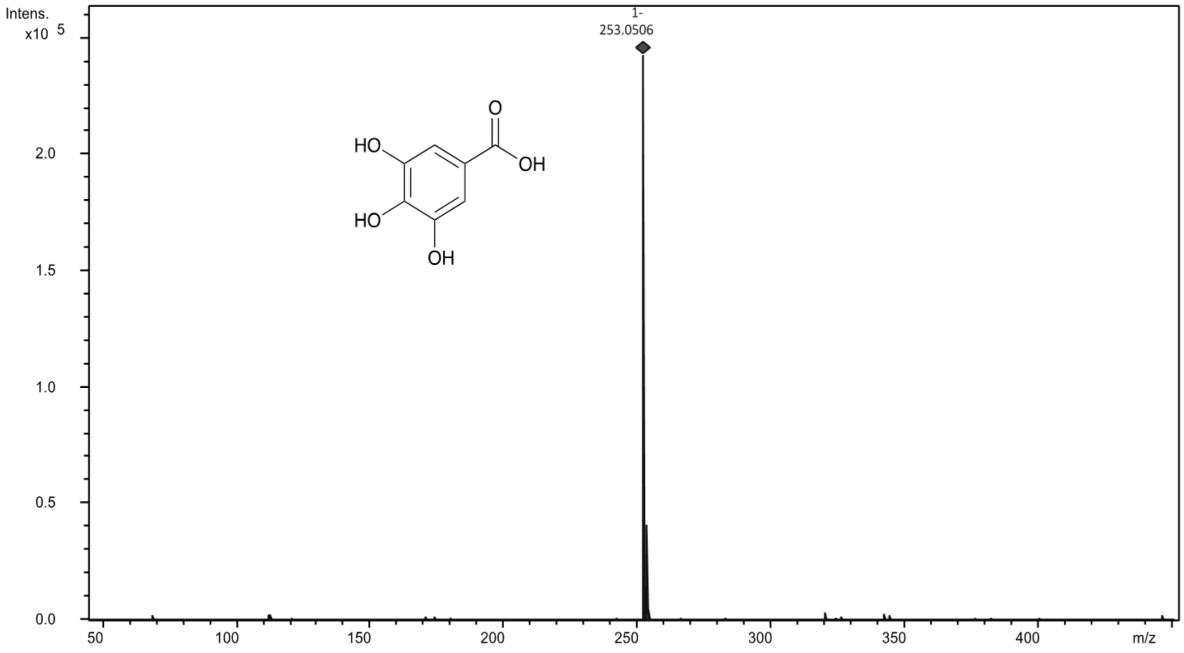

Figure S1. Peak A, found in fermented Jamaican cherry juice with a negative HR-ESI-TOF-MS m/z 235.0506 [M+H]$^-$.

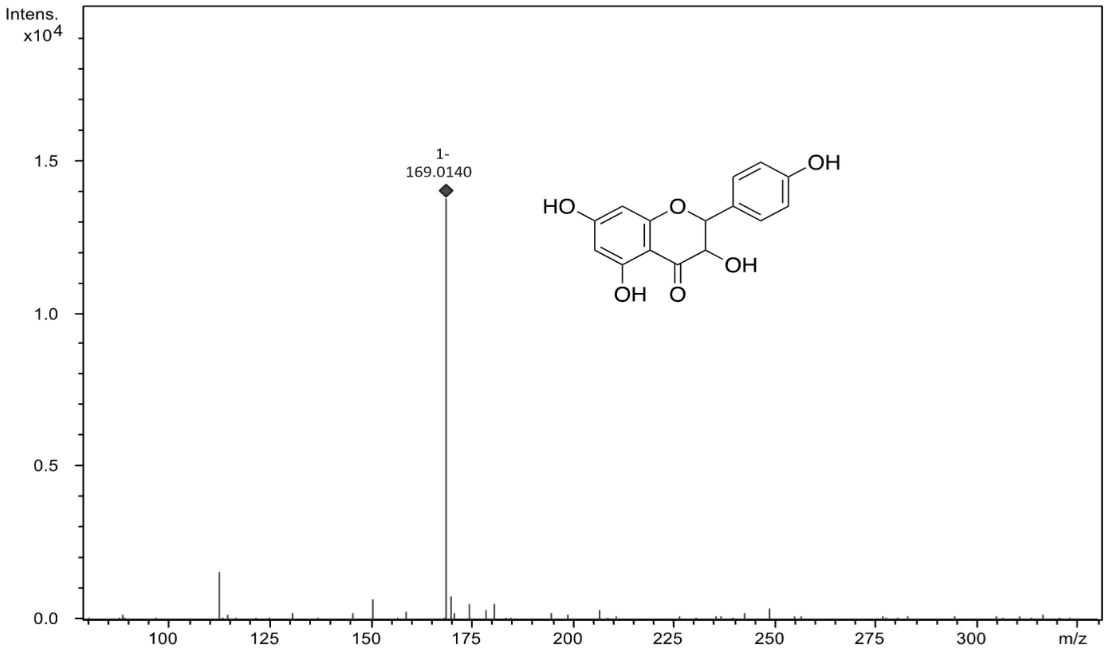

Figure S2. Peak B, found in fermented Jamaican cherry juice with a negative HR-ESI-TOF-MS m/z 169.0140 [M+H]$^-$.

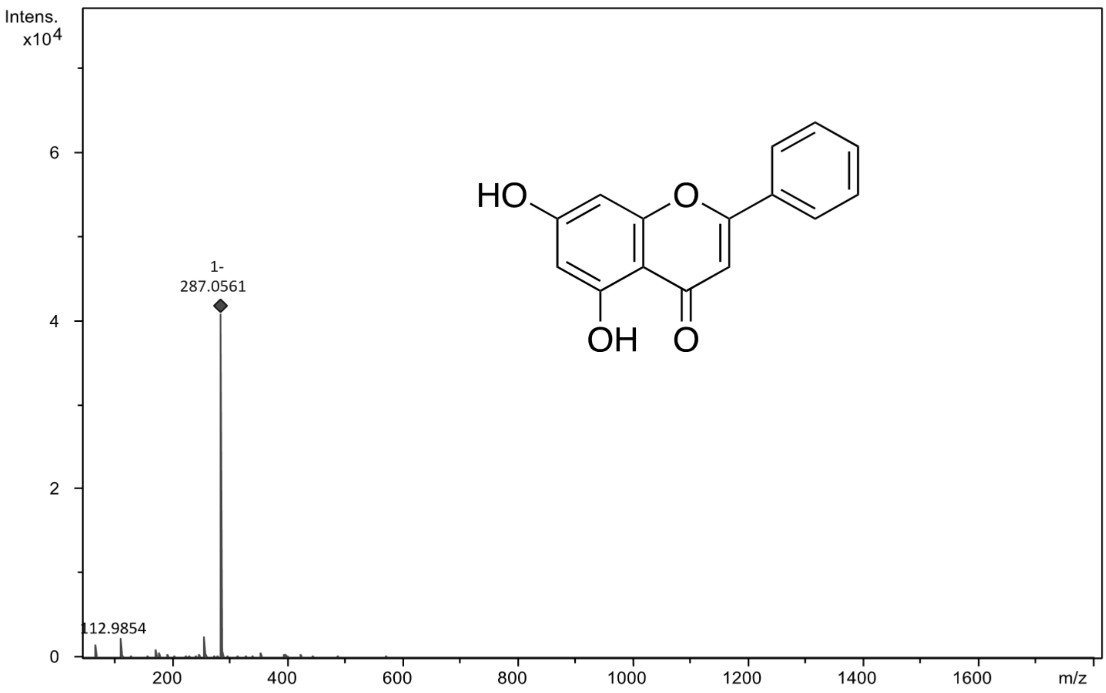

Figure S3. Peak C, found in fermented Jamaican cherry juice with a negative HR-ESI-TOF-MS m/z 287.0561 $[M+H]^-$.
